# Bixin from *Bixa orellana* L.: Analytical Method Validation, Physicochemical Characterization, and Selective Antifungal Activity against *Candida* spp. 

**DOI:** 10.1021/acsomega.5c04986

**Published:** 2025-08-15

**Authors:** Deborah Fernandes Rodrigues, Marina Vieira, Marcel Alexandre Formaggio Moraes Júnior, Rodrigo Michelini Oliveira Thomasi, Ruben Dario Sinisterra Millán, Eliana Faria Garcia, Renê Oliveira Couto, William Gustavo Lima, Magna Cristina Paiva, Carlos Eduardo Matos Jensen

**Affiliations:** † 573437 Universidade Federal de São João del-Rei (UFSJ), Campus Centro-Oeste Dona Lindu, R. Sebastião Gonçalves Coelho 400, Divinópolis, MG 35501-296, Brazil; ‡ Departamento de Química do ICEx, Universidade Federal de Minas Gerais, Av. Presidente Antônio Carlos, 6627, Belo Horizonte, MG 31270-901, Brazil; § Faculdade de Ciências Médicas de Minas Gerais, Alameda Ezequiel Dias, 275, Belo Horizonte, MG 30130-110, Brazil; ∥ Faculdade de Saúde Santa Casa, 601173 Grupo Santa Casa de Belo Horizonte, Av. dos Andradas, 2688, Belo Horizonte, MG 30110-005, Brazil

## Abstract

Bixin is a lipophilic apocarotenoid abundant in the aril of *Bixa orellana* L. seeds. Widely used as a natural colorant in food, textile, and cosmetic industries, its pharmacological potential remains underexplored. This study aimed to extract and purify bixin, characterize its physicochemical properties, develop and validate an HPLC method for quantification, and evaluate its antifungal activity, cytotoxicity, and selectivity *in vitro*. Extraction was performed by Soxhlet using hexane and chloroform, followed by recrystallization in acetone. Characterization (TG/DTA, FTIR, and ^1^H NMR) confirmed predominance of the 9’-*cis* isomer. The HPLC method (λ=470 nm) was validated per ICH Q2­(R2), showing specificity, linearity (R^2^ = 0.9993), low LOD/LOQ (0.638 and 1.934 μg mL^–1^), and high precision and accuracy (RSD < 2%; recovery 99.4–100.8%). Antifungal assays against eight *Candida* strains revealed moderate activity, with MICs from 2 to 256 μg mL^–1^. The best results were observed for *C. glabrata* and *C. tropicalis* (MIC = 2 and 4 μg mL^–1^). Bixin also inhibited virulence factors in*C. albicans* Cytotoxicity on Vero cells showed a CC_50_ of 30.71 μg mL^–1^, with selectivity indices of 15.36 and 7.68 for*C. glabrata* and*C. tropicalis*, respectively. These results support bixin’s potential as a natural antifungal agent.

## Graphical abstract

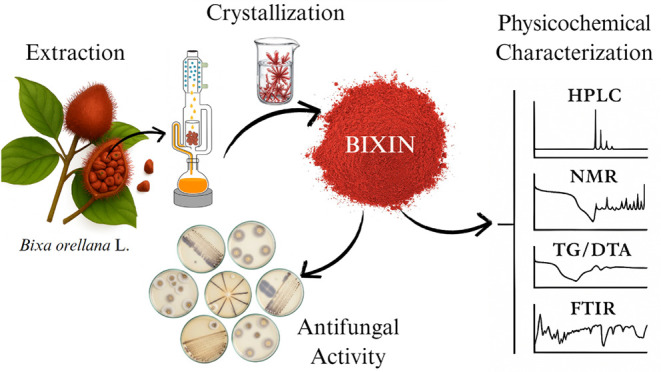

## 1 Introduction

Plants are widely recognized for their capacity to produce a broad array of secondary metabolites, many of which have been traditionally employed in the treatment of various diseases. Numerous natural products display significant biological and pharmacological activities, serving as important leads for the development of novel therapeutic agents.^1,2^

In this context, bixin, an apocarotenoid pigment extracted from the surface of *Bixa orellana* L. seeds, has attracted considerable scientific interest due to its diverse chemical, biological, and medicinal properties.^3^ Studies have demonstrated its potential activities, including provitamin A activity,^4^ antioxidant,^5–7^ anti-inflammatory,^8^ antitumor,^8,9^ hypoglycemic,^10^ hypolipidemic,^11^ and antimicrobial effects.^5,7,12^

Bixin comprises approximately 80% of the total carotenoids in annatto seeds, followed by norbixin and phenolic compounds. Its solubility profile enables the preparation of both lipophilic and hydrophilic extracts with distinct applications. Consequently, annatto extracts are widely used in food, cosmetic, and pharmaceutical formulations.^13^

Among natural colorants, bixin stands out for its physicochemical stability, vivid color, and ease of extraction. These advantages, combined with the widespread cultivation of annatto in tropical regions of South America, India, and Africa, underscore its industrial relevance.^14,15^

Despite its broad use as a coloring agent, further investigation into bixin’s physicochemical and pharmacological properties is necessary to support its potential as a bioactive compound. Moreover, given the global rise in fungal infections and increasing resistance to available antifungal agents, there is a pressing need to identify novel antifungal candidates from natural sources.^16,17^

Therefore, this study aims to (i) isolate, purify, and characterize the physicochemical properties of bixin; (ii) develop and validate a rapid, simple, and reproducible HPLC method for its quantification; (iii) evaluate its *in vitro* antifungal activity against *Candida* spp.; and (iv) assess its cytotoxicity and selectivity *in vitro*.

## 2 Results and Discussion

### 2.1 HPLC Method for Bixin Analysis

To propose a method for the identification and quantification of bixin using HPLC, we attempted to reproduce the analytical conditions described by Scotter et al. (1995), which are widely cited in the literature. Under the same working conditions, bixin was identified after 22.5 min of analysis, in contrast to the retention time reported, which was 5.4 min.^18^

In this context, to reduce the analysis time for bixin, we opted to increase the proportion of acetonitrile in the mobile phase, changing from the 65:35 (v/v) mixture of acetonitrile and 0.4% acetic acid used by Scotter et al. to an 80:20 (v/v) mixture of acetonitrile and 2% acetic acid. The working temperature of the chromatographic column, a C18 (250 mm × 4.6 mm × 5 μm) column in both methods, was also raised from 35 to 40 °C, which enhanced elution efficiency while reducing system pressure, allowing for the use of a slightly higher flow rate (1.2 mL min^–1^ compared to 1.0 mL min^–1^ in the original method).

The injection volume, not reported by Scotter et al., was defined as 20 μL in the new protocol. Although the literature suggests the widespread use of diode array detectors (DAD), the availability of equipment in the laboratory led to the use of a UV–vis spectrophotometer, with detection set at 470 nm, as opposed to 435 nm in the previous method.

With the new HPLC analytical method, it was possible to detect and quantify bixin with a retention time of 5.950 min. Figures 1 and 2 present chromatograms obtained from the analysis of the 25 μg mL^–1^ bixin standard and the sample at the same concentration.

**Figure 1:**
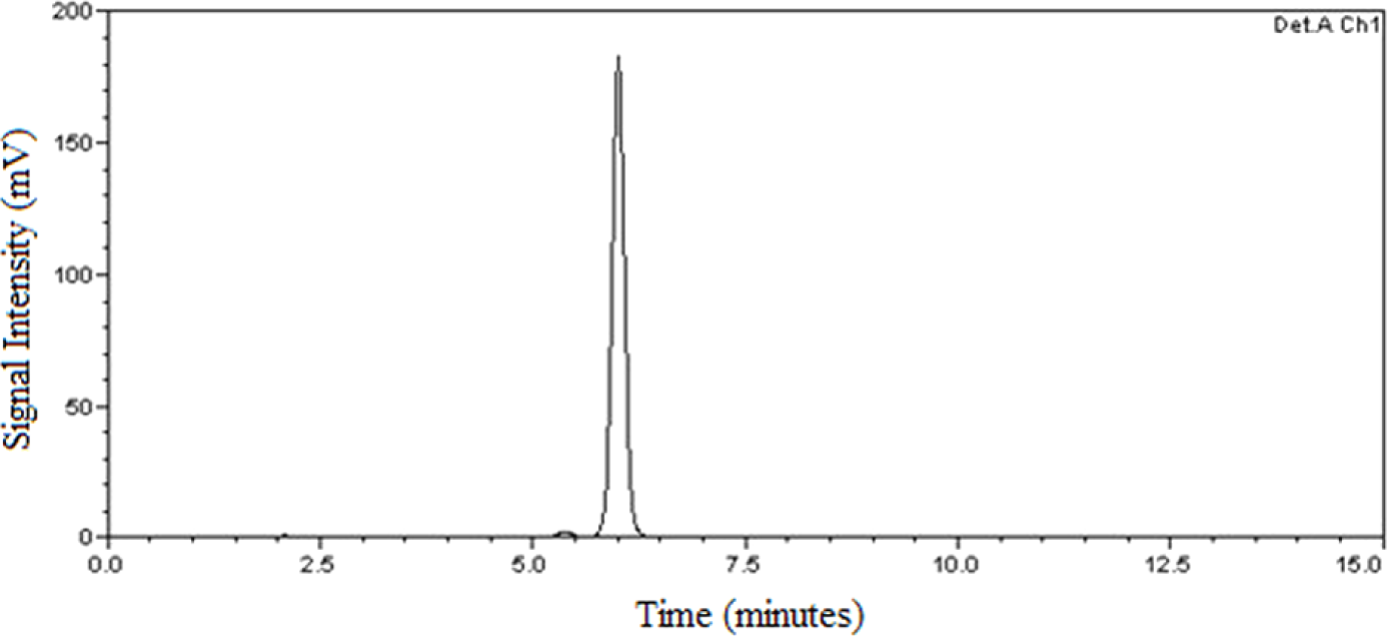
Chromatogram of the bixin standard. Chromatographic conditions: C18 column (250 mm × 4.6 mm, 5 μm particle size), detector wavelength: 470 nm, column temperature: 40 °C, mobile phase: acetonitrile and 2% acetic acid in an 80:20 v/v, under isocratic conditions, flow rate: 1.2 mL min^–1^.

**Figure 2:**
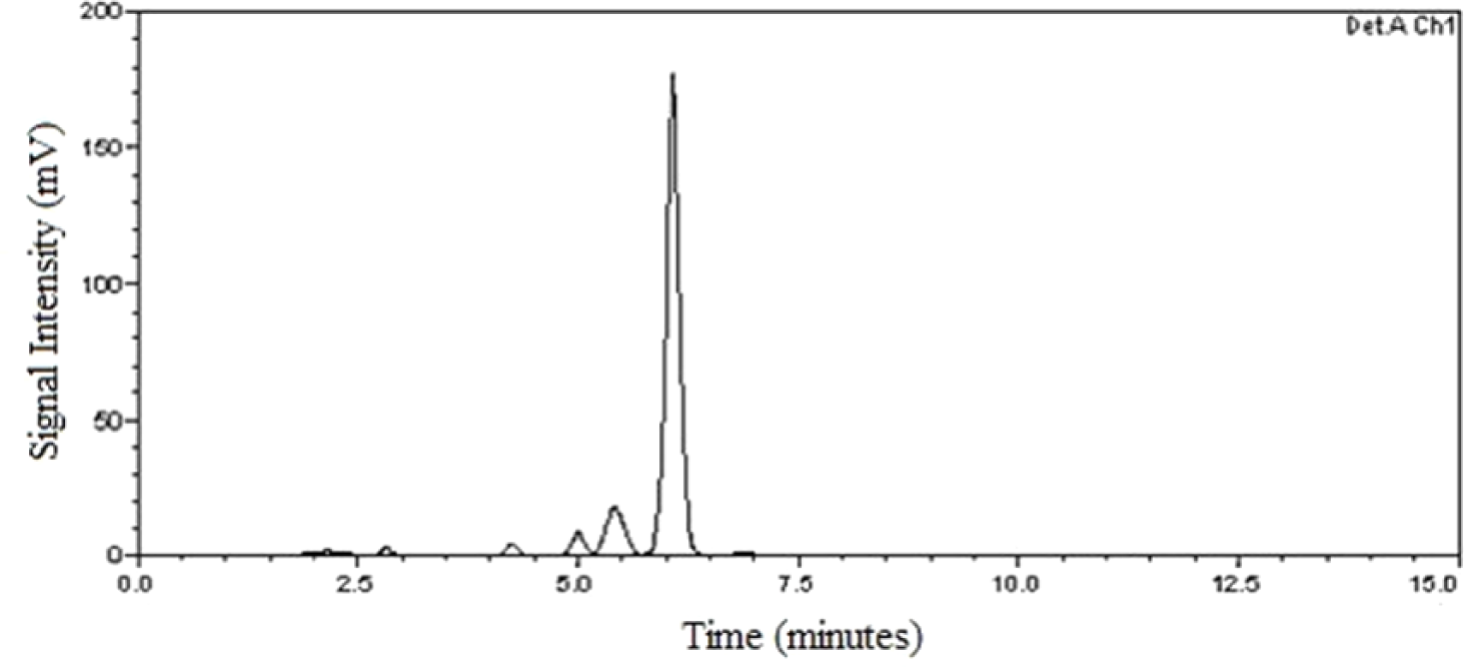
Chromatogram of the purified bixin sample obtained by extraction and recrystallization. Chromatographic conditions: C18 column (250 mm × 4.6 mm, 5 μm particle size), UV–vis detector wavelength: 470 nm, column temperature: 40 °C, mobile phase: acetonitrile and 2% acetic acid in an 80:20 v/v ratio, under isocratic conditions, flow rate: 1.2 mL min^–1^.

To ensure that the new analytical method provides reliable and interpretable data, the proposed method was validated, and the results of the system suitability test are shown in Table 1.

**Table 1:** Results of the System Suitability Test for the Validation of Bixin Quantification Using the Proposed Method

Parameters	Results	Specification
Number of theoretical plates	17,341	>2,000
Symmetry factor	1.09	<2.0
RSD (Relative Standard Deviation)	0.59	<2.0
Resolution	—	>2.0

The results of the tests performed suggest that the developed method met the specifications, demonstrating that the system is compliant for conducting the analyses. By analyzing the retention times and peak areas (T), the method’s ability to detect each substance independently was confirmed.

Only polyvinylpyrrolidone (PVP) was detected by the method, with a retention time of 1.177 min. The resolution (R), or separation factor, between the peaks corresponding to PVP and bixin was 4.29. This indicates that the substances were satisfactorily separated, and each peak corresponds to a single substance.

The linearity analysis indicated that linear regression could be calculated using the least-squares method (LSM), which yielded the representative linearity equation (y = 42309 x + 87225). From the linear regression analysis, the determination coefficient (R^2^ = 0.9993) was calculated, ensuring a strong correlation or linearity in the curve within the range of 12.5 μg mL^–1^ to 75.0 μg mL^–1^. The results for precision, demonstrated through repeatability and intermediate precision, and for accuracy are presented in Table 2.

**Table 2:** Repeatability, Intermediate Precision, and Accuracy of the Spectrophotometric Analytical Method for the Determination of Bixin (*N* = 3)

Repeatability (Day 1)			Repeatability (Day 2)		
Concentration (μg mL^–1^)	Recovery (%)	CV	Concentration (μg mL^–1^)	Recovery (%)	CV
25.0	99.5	1.64	25.0	100.2	1.89
50.0	100.8	1.07	50.0	100.4	2.46
75.0	99.7	0.54	75.0	100.3	0.67
25.0	100.1	0.76	25.0	99.9	1.4
50.0	99.4	0.99	50.0	100.2	1.5
75.0	99.9	1.17	75.0	99.9	0.8

Based on the coefficient of variation (CV) values, it can be ensured that there is no significant variability, as the values are relatively close, with variations below 5%,^19^ which confirms the repeatability of the methods. Considering the average CV results obtained on different days of analysis and by different analysts, the method can also be considered reproducible.

Furthermore, the recovery percentages ranged from 99.4% to 100.8% in all analyzed samples, demonstrating the accuracy of the proposed method. The calculated detection and quantification limits were 0.638 μg mL^–1^ and 1.934 μg mL^–1^, respectively.

### 2.2 Extraction Yields and Physicochemical Properties of Bixin

Bixin (Figure 3) was successfully extracted from *B. orellana* L. seeds and subsequently purified by recrystallization, yielding orange-red crystals with high purity. The identity and chemical integrity of the compound were confirmed by HPLC, following method validation in accordance with ICH Q2­(R2) guidelines. Structural and physicochemical characterization was further carried out using thermal analysis (TG/DTA), attenuated total reflectance Fourier transform infrared (ATR-FTIR) spectroscopy, and proton nuclear magnetic resonance (^1^H NMR) spectroscopy. These analyses confirmed the purity, thermal stability, and molecular structure of the purified bixin.

**Figure 3:**
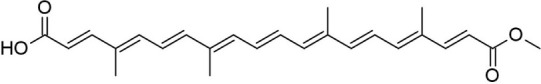
Chemical structure of bixin.

Quantitative analysis of the recrystallized product was carried out using an HPLC method previously developed and validated by our group as part of this study. The chromatographic conditions included a C18 column (250 mm × 4.6 mm, 5 μm), isocratic elution with acetonitrile and 2% acetic acid (80:20 v/v), a flow rate of 1.2 mL min^–1^, and UV–vis detection at 470 nm. The purity of the bixin sample, purified and crystallized, was determined to be 95.86%, confirming the efficiency of the extraction and purification process as well as the robustness of the analytical method.

The thermal behavior of the compound was evaluated by simultaneous thermogravimetric (TG) and differential thermal analysis (DTA), and the resulting curves are shown in Figure 4. These analyses provide insights into the thermal stability, decomposition profile, and possible phase transitions of the molecule.

**Figure 4:**
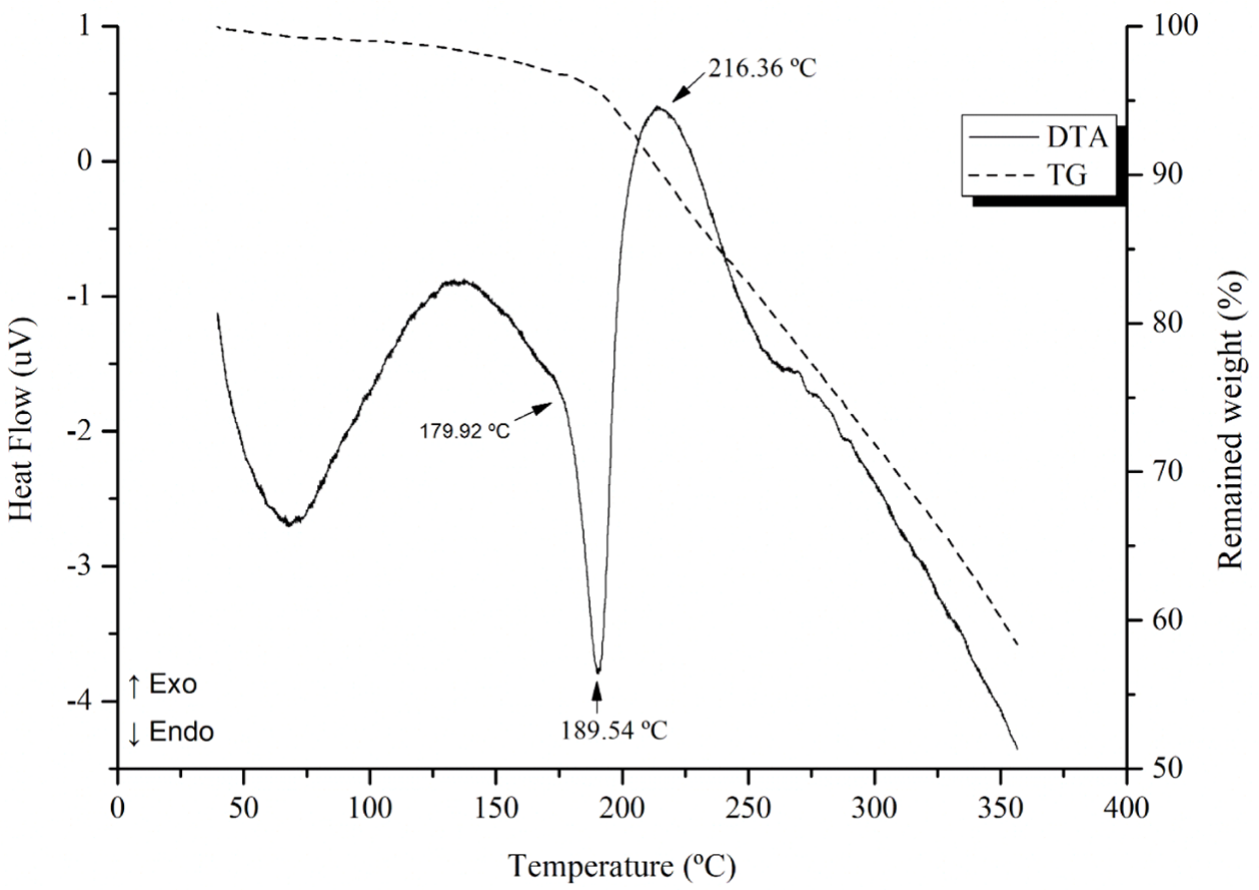
TG and DTA curves of bixin obtained under a dynamic N_2_ atmosphere, with a flow rate of 50 mL·min^–1^ and a heating rate of 10 °C min^–1^.

The thermal analysis of bixin determined an endothermic peak appears at 189.54 °C, with the onset of melting at 179.92 °C and completion at 216.36 °C. Similarly, in the TG curve, decomposition begins simultaneously with the melting process. The observed melting point aligns with reported values for α-bixin (189.5–190.5 °C), whereas *trans*-bixin melts at a higher range (204–206 °C). These findings indicate that the extracted compound is predominantly in the α-form, with minimal or no presence of the *trans* isomer in the analyzed sample.^20–22^

The Fourier-transform infrared (FTIR) spectrum, presented in Figure 5, reveals the characteristic vibrational modes of the functional groups present in the molecule, aiding in the confirmation of its structural features and purity. Furthermore, the ^1^H nuclear magnetic resonance (^1^H NMR) spectrum, shown in Figure 6, offers detailed information on the hydrogen framework of the bixin molecule, corroborating the conjugated polyene chain and the substitution pattern along the aliphatic backbone. Together, these complementary techniques provide a robust physicochemical profile of bixin, contributing to a better understanding of its structural integrity, stability, and potential applicability in pharmaceutical formulations.

**Figure 5:**
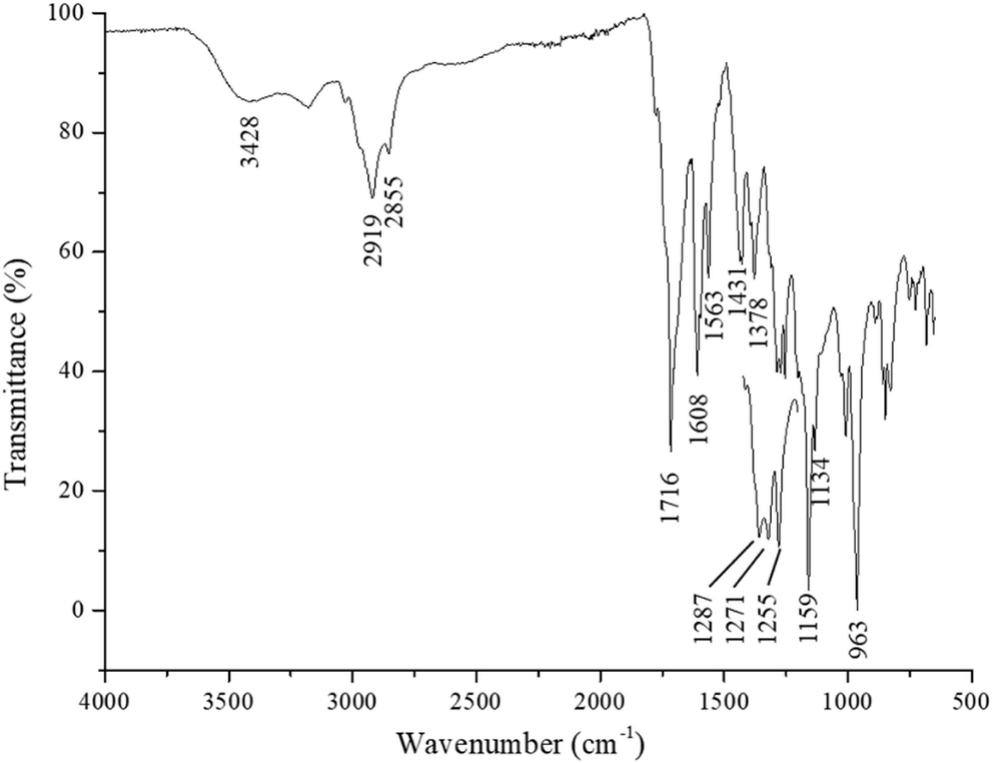
Fourier transform infrared (FTIR) spectrum of bixin.

**Figure 6:**
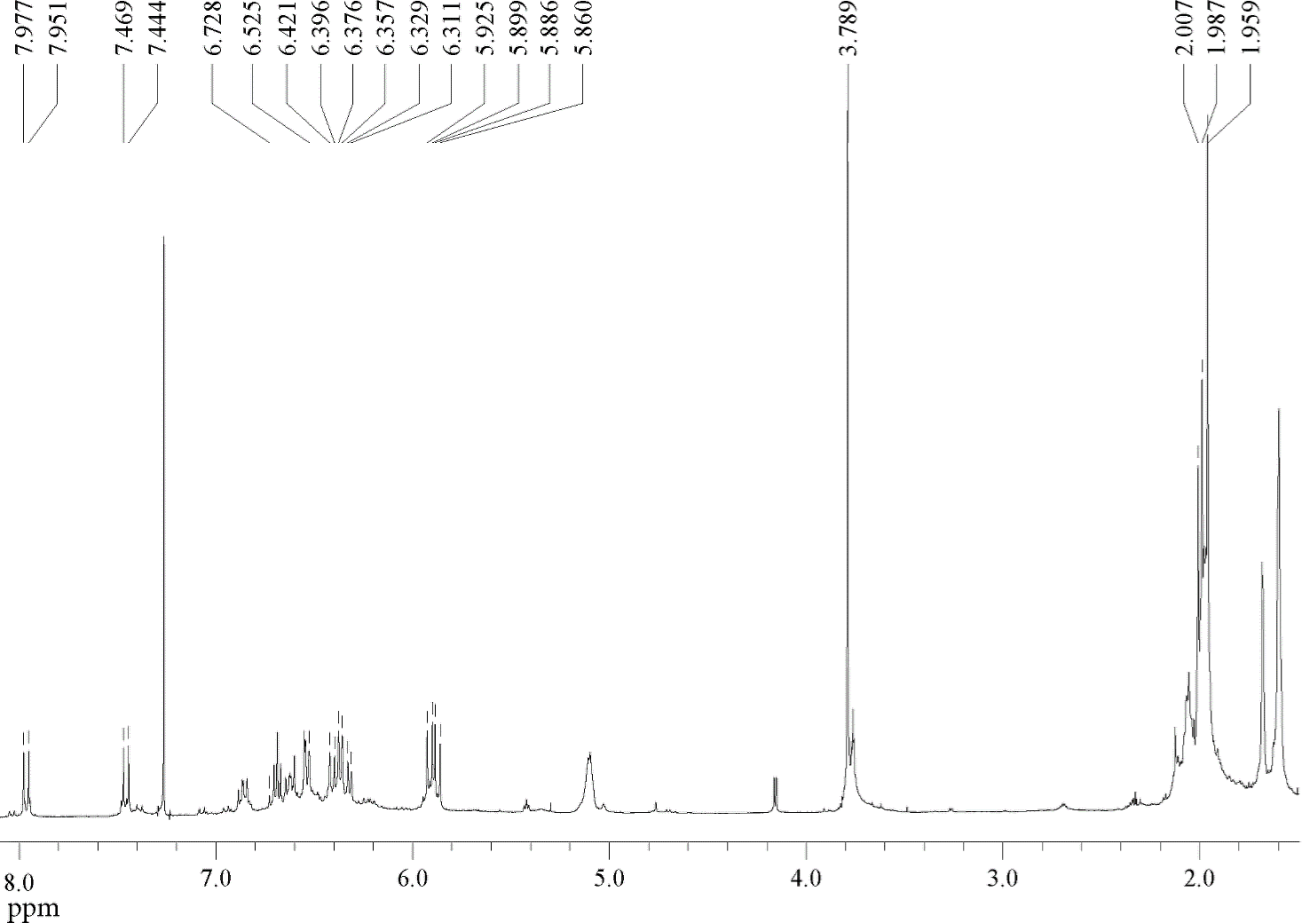
^1^H NMR spectrum in CDCl_3_ (400 MHz) for bixin at a temperature of 27 °C.

FTIR spectroscopy plays a pivotal role in characterizing the functional groups and molecular structure of α-bixin, a carotenoid extracted from *B. orellana* L. The FTIR spectrum reveals several diagnostic absorption bands. A strong absorption band at 1716 cm^–1^ is attributed to the stretching vibration of the ester carbonyl group (C = O), confirming the presence of an ester moiety, which is essential to α-bixin’s structural integrity.^23^ The conjugated alkene system is evidenced by multiple C = C stretching vibrations observed at 1431, 1563, and 1608 cm^–1^, reflecting the extended π-electron system that underlies its chromophoric and antioxidant properties.^23,24^

The ester functionality is further confirmed by the symmetric and asymmetric stretching vibrations of the C–O–C group, detected at 1255 cm^–1^ and 1159 cm^–1^, respectively.^23,25^ Additionally, the band at 1378 cm^–1^ corresponds to the bending vibration of methyl (−CH_3_) groups, while the aliphatic C–H stretching vibrations of methylene and methyl groups^23–25^ appear near 2919 and 2855 cm^–1^. A broad absorption around 3428 and 3177 cm^–1^ is assigned to the O–H stretching of hydroxyl groups, possibly associated with carboxylic acid or hydrogen bonding interactions.^23,25^

Importantly, the band at 963 cm^–1^ is characteristic of *cis*-isomers of carotenoids, which is relevant for identifying the geometric configuration of α-bixin in its natural form.^26^ Altogether, these spectral features validate the presence of conjugated alkenes, ester linkages, hydroxyl functionalities, and *cis*-configuration, corroborating the molecular structure of α-bixin as previously proposed in literature.

The analysis of bixin by ^1^H NMR spectroscopy, performed in deuterated chloroform, enabled the assignment and identification of hydrogen signals along the polyene chain and the terminal functional groups of the molecule. The experimental data are in agreement with previously reported chemical shift values for natural bixin isolated from *B. orellana* L. seeds and are consistent with the predominance of *cis* geometric isomerism at the 9’ position -corresponding to the 9’-*cis*-bixin (9’Z) configuration.^27–29^

The signals of H8’ (δ 7.964 ppm, doublet, J = 13 Hz) and H8 (δ 7.456 ppm, doublet, J = 12.5 Hz) are characteristic of vicinally coupled vinylic protons through a *trans*-configured double bond, as evidenced by their large coupling constants (J ≈ 13 Hz). This pattern is in accordance with literature reports indicating the presence of extended *trans* segments within the polyene backbone of bixin, even in molecules predominantly adopting a *cis* configuration.^30,31^ The relatively downfield chemical shift of H8’ can be attributed to deshielding effects caused by proximity to electron-withdrawing groups or additional unsaturations.^32^ Similarly, the H7’ (δ 5.912 ppm) and H7 (δ 5.873 ppm) doublets, also with J = 13 Hz, support the presence of alternating *trans*-configured units within the conjugated chain.^27,29^

Despite these localized *trans* characteristics, the overall NMR spectral pattern, in conjunction with comparative reference spectra, confirms the global *cis* configuration at position 9’, as expected for the naturally occurring form. The 9’ position is recognized as the primary stereogenic site of bixin, and the Z configuration at this site is thermodynamically favored during natural biosynthesis. It remains the dominant isomer in seed extracts not subjected to thermal or photochemical isomerization.^18,33^

The observation of multiple signals in the δ 6.3–6.7 ppm region, assigned to protons H10, H10’, H11, H11’, H12, H12’, H14, H14’, H15, and H15’, reflects a highly conjugated polyene system. The complex splitting patterns indicate the involvement of both vicinal and long-range couplings. The doublet of doublets for H11’ (δ 6.863 ppm, J = 8.0 and 7.6 Hz) is particularly suggestive of a deshielded proton flanked by double bonds in potentially different configurations, consistent with a conjugated *cis* environment within the polyene chain.^27,32^

Additionally, the singlets observed at δ 1.959 ppm (H19 and H19’), δ 2.007 ppm (H20’), and δ 1.987 ppm (H20) correspond to methyl groups bonded to sp^2^ -hybridized carbons, a typical feature in conjugated carotenoid systems.^28,31^ The presence of distinct signals for H19/H19’ and H20/H20’ further supports the asymmetry of the molecule, which arises from the presence of a free carboxylic acid at one terminus and a methyl ester at the opposite end. The ester methyl proton resonates as a singlet at δ 3.789 ppm, which is consistent with values reported for structurally related systems.^27–29^

Comparison with the data reported by Rehbein et al. (2007) confirms the accuracy of the signal assignments. Their LC-NMR investigation yielded nearly identical chemical shifts for the 9’-*cis* isomer, supporting the conclusion that no isomerization occurred during sample handling or purification. These findings are further corroborated by the earlier work of Barber et al. (1961), who differentiated bixin stereoisomers based on NMR signatures and established that the natural form is monocis (*cis*-9’), in contrast to the fully *trans* form that can be generated via thermal or photochemical isomerization.^28,30^

The ^1^H NMR data obtained are consistent with the proposed structure of bixin in the (9’Z) configuration, featuring a predominantly *trans* polyene backbone with a key *cis* double bond at position 9’. This structural confirmation underscores the utility and reliability of NMR spectroscopy for the elucidation of natural pigment structures and the discrimination of geometric isomers.

### 2.3 *In Vitro* Antifungal Susceptibility Testing

The results of the qualitative screening test for the antifungal activity of purified bixin are presented in Table 3.

**Table 3:** Inhibition Zones (mm), Minimum Inhibitory Concentrations (MICs), Minimum Fungicidal Concentrations (MFCs) of Bixin against *Candida* spp., and the Selectivity Index of Bixin with Respect to Mammalian Renal Cells^a^

	Inhibition Zones (mm, disk diffusion test)					
Species	Bixin (10 μg)	Bixin (25 μg)	Bixin (190 μg)	MIC	MFC	Selectivity Index-SI
*C. albicans* ATCC 26790	-	-	-	256	>1024	0.12
*C. albicans* ATCC 14053	-	-	-	64	>1024	0.48
*C. albicans* ATCC 18804	-	-	-	16	>1024	1.92
*C. glabrata* ATCC 2001	-	-	-	2	1024	15.36
*C. krusei* ATCC 6258	-	-	-	32	>1024	0.96
*C. krusei* ATCC 34135	11	10	10	64	>1024	0.48
*C. parapsilosis* ATCC 22019	-	-	-	64	>1024	0.48
*C. tropicalis* ATCC 28707	-	-	-	4	1024	7.68

a MIC: Minimum Inhibitory Concentration; MFC: Minimum Fungicidal Concentration. MIC and MFC are expressed in μg mL^–1^. All values represent the mean of three replicates. (−): No inhibition zone was observed.

Initially, in the disk diffusion test, a possible antifungal activity of bixin was observed against *C. krusei* ATCC 34135 which is particularly relevant given that this species is known to be resistant to azoles and polyenes.^34–36^ Indeed, as also reported in several studies,^59–62^ resistance to fluconazole was here evidenced by the absence of inhibition zones in the assays performed for the nonalbicans *Candida* species.

Studies evaluating the antifungal activity of purified bixin are not found, in contrast to the use of extracts. Irobi et al. (1996) and Fleischer et al. (2003) demonstrated antifungal activity against *C. utilis* and *Aspergillus niger* and *C. albicans*, respectively, using 95% ethanol extract (5 mg mL^–1^) of *B. orellana* L. Furthermore, Poma-Castillo et al. (2018) reported that the antifungal activity of ethanol extract is proportional to the tested concentrations.^37–39^

Considering the qualitative method used in this study (disk diffusion), and the results found for purified bixin, some factors must be considered. For example, the absence of antifungal activity, suggested by the absence of an inhibition zone, may be associated with the chemical characteristics of bixin, such as lipophilicity, which may have compromised the diffusion of the compound in the culture medium and consequently impeded its potential activity against the tested fungi. Additionally, it should be noted that low solubility, difficulty in incorporation and diffusion in culture media, and/or instability of dilutions of certain compounds, particularly those of plant origin, further limit the applicability of these tests.^40,41^

To exclude potential interferences related to the characteristics of bixin, a quantitative broth microdilution test was performed, a technique considered the gold standard.^42^ The MICs and MFCs are shown in Table 3. According to Mbaveng et al. (2015), the antifungal activity of a phytochemical is defined as significant when the MIC is less than 10 μg mL^–1^, moderate when 10 μg mL^–1^ < MIC < 100 μg mL^–1^, and low when MIC exceeds 100 μg mL^–1^. Thus, bioactivity of purified bixin against the *Candida* spp. included in this study was observed.^43^

Moderate bioactivity of purified bixin was noted for most of the *Candida* strains (MICs 16–64 μg mL^–1^), and the differences in MIC may be related to the microbiological characteristics of the strains, such as antifungal resistance. For example, *C. albicans* ATCC 18804 is reported to have cross-resistance to amphotericin B,^44^ while *C. albicans* ATCC 14053 is resistant to fluconazole.^45^ In this same context, the finding of moderate activity against *C. krusei* ATCC 6258 and 34135 and *C. parapsilosis* ATCC 22019 should be highlighted, as these species are known to be resistant to clinically relevant antifungals.^36,46^

On the other hand, a noteworthy finding was the significant activity of bixin against *C. glabrata* ATCC 2001 and *C. tropicalis* ATCC 28707 (Table 3), the latter being intrinsically resistant to amphotericin B, one of the most commonly used antifungals in clinical practice. *C. glabrata*, now *Nakaseomyces glabrata* ^47^ frequently causes mucosal and disseminated candidiasis, with higher mortality rates observed among infected patients.^48–51^ Meanwhile, *C. tropicalis* exhibits a wide range of virulence factors, has the ability to form biofilms,^52–54^ and has been reported as a leading cause of invasive candidiasis in neutropenic patients.^55^ Additionally, these species have shown increasing resistance to azoles in clinical isolates.^56^

Indeed, many studies using phytocompounds have been conducted against these species, considering their increased prevalence and resistance to antifungals. β-citronellol, found in the essential oil of various plants^57^ and ethanol extract of *Persea americana* leaves,^58^ were tested, but the obtained MICs were greater than 400 μg mL^–1^. Similarly, for curcumin, MICs of ≥ 0.4 mg mL^–1^ were obtained for *C. tropicalis* and *C. glabrata*,^59,60^ and the authors concluded that this compound could be an interesting alternative or complementary option for the treatment of candidiasis. Thus, our findings point to the significant therapeutic potential of bixin in filling the observed gap between antifungals.

In addition to fungistatic activity, the fungicidal potential of bixin (MFC) was evaluated and is presented in Table 3. Considering the species resistant to currently available antifungals, the fungicidal activity of bixin is even more desirable, as eliminating the fungal load is crucial to prevent relapses in treatment, especially in immunocompromised patients.^61^ However, it appears that bixin, at least in the form tested in this study, does not exhibit fungicidal activity (MFC ≥ 1.024 μg mL^–1^).

#### 2.3.1 Activity of Purified Bixin on *C. Glabrata* ATCC 2001 and *C. tropicalis* ATCC 28707

The death curve allows for the assessment of the lethal action rate of a specific concentration of a compound with potential antimicrobial activity against the microorganism, as well as revealing the relationship between concentration and activity over time.^62–65^

The results of the death curves for *C. glabrata* ATCC 2001 and *C. tropicalis* ATCC 28707, which had the lowest MICs and MFCs of bixin determined (Table 3), using the MFC of bixin (1024 μg mL^–1^), were plotted in graphs expressing Log_10_ CFU mL^–1^ values over time and are represented in Figures 7.

**Figure 7:**
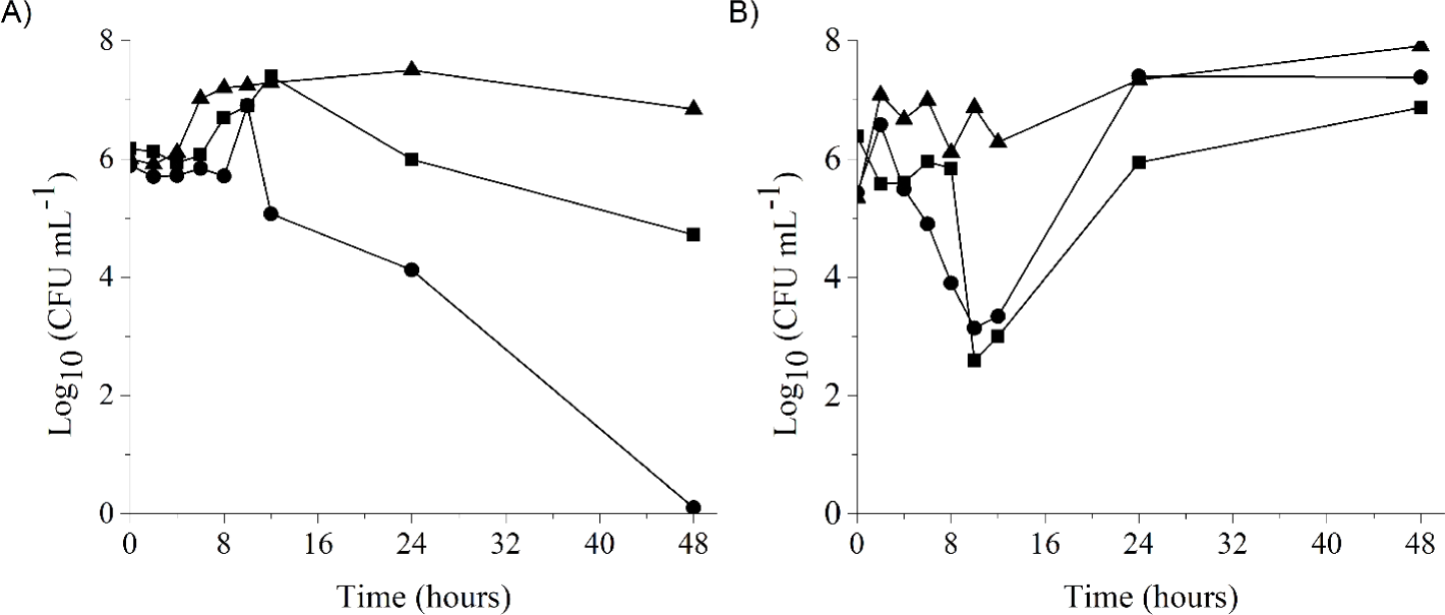
Graphical representation of the fungal death curve (Log_10_ CFU mL^–1^) for *C. glabrata* ATCC 2001 (A) and *C. tropicalis* ATCC 28707 (B) under the action of bixin (1024 μg mL^–1^, ■), nystatin (positive control, 0.25 μg mL^–1^, ●), and no antifungal (growth control, ▲) over time.

The action of bixin on the studied *Candida* species begins after 4 h of exposure, and after 8 and 10 h, the activity profile resembles that of the positive control, suggesting that bixin acts on fungal cells similarly to nystatin, a broad-spectrum antifungal used in the topical treatment of oral, mucosal, and cutaneous infections.^66^

The most pronounced activity of bixin, particularly against *C. tropicalis*, was observed after 10 h of exposure. At this point in the bixin death curve, there was a reduction of approximately 4 Log_10_ CFU mL^–1^ compared to the initial inoculum, while nystatin showed a significantly smaller reduction (around 2 Log_10_ CFU mL^–1^). This indicates good activity of bixin in inhibiting the growth and viability of fungal cells for this species. Although *C. tropicalis* resumed growth similarly in the presence of both bixin and nystatin after 12 h of the experiment, the end point growth for bixin was lower than that observed for nystatin when compared to the untreated control.

For *C. glabrata* ATCC 2001, the reduction in cellular growth at the end point for nystatin (a reduction of 6 Log_10_ CFU mL^–1^) was greater than that for bixin (approximately 1.5 Log_10_ CFU mL^–1^). However, compared to the untreated control, bixin reduced the growth of *C. glabrata* ATCC 2001 by 2 Log_10_ CFU mL^–1^, which corresponds to about a 70% reduction in the number of viable cells.

It is noteworthy that the variation observed at the initial points of the death curve may be associated with the microorganism’s adaptation or the antifungal compounds’ mechanisms of action, as this phenomenon is also observed for the positive control. Therefore, it can be suggested that bixin is a potential fungistatic compound against various *Candida* species, particularly against *C. glabrata* and *C. tropicalis*, but with fungicidal action only at elevated concentrations. However, further studies may reveal its use as a fungicide at lower concentrations, for example, in combination with other antifungals, demonstrating synergistic activity.

#### 2.3.2 Anti-Virulence Factor Activity of Purified Bixin

The yeast-to-hypha transition is a crucial virulence factor in *Candida* species, playing a significant role in the success of infections, resistance to medications, and evasion of phagocytosis by macrophages. Almeida et al. (2008) demonstrated that *C. albicans* in its hyphal form uses the adhesin Als3 to bind to ferritin within epithelial cells, acquiring iron for its growth, which is essential for fungal cell viability.^67^

One of the most critical actions for antifungals to exhibit high clinical efficacy is their ability to inhibit or combat virulence factors in *Candida* spp.^61^ To evaluate the action of bixin on this virulence event, assays were conducted against *C. albicans* ATCC 18804, considering its inherent capacity for hyphal formation.^68^ As shown in Figure 8, the effect of bixin on fungal cells was dose-dependent. At a concentration of 1/2 MIC (8 μg mL^–1^), the inhibition of the transition was not complete, as some filamentous structures were still observed. However, at a MIC of 16 μg mL^–1^, the inhibition of the transition became more effective, with a complete absence of hyphae observed at 2x MIC of bixin (32 μg mL^–1^). Fluconazole completely inhibited the yeast-to-hypha transition, and untreated cells formed large amounts of hyphal structures, thus validating the experimental conditions.

**Figure 8:**
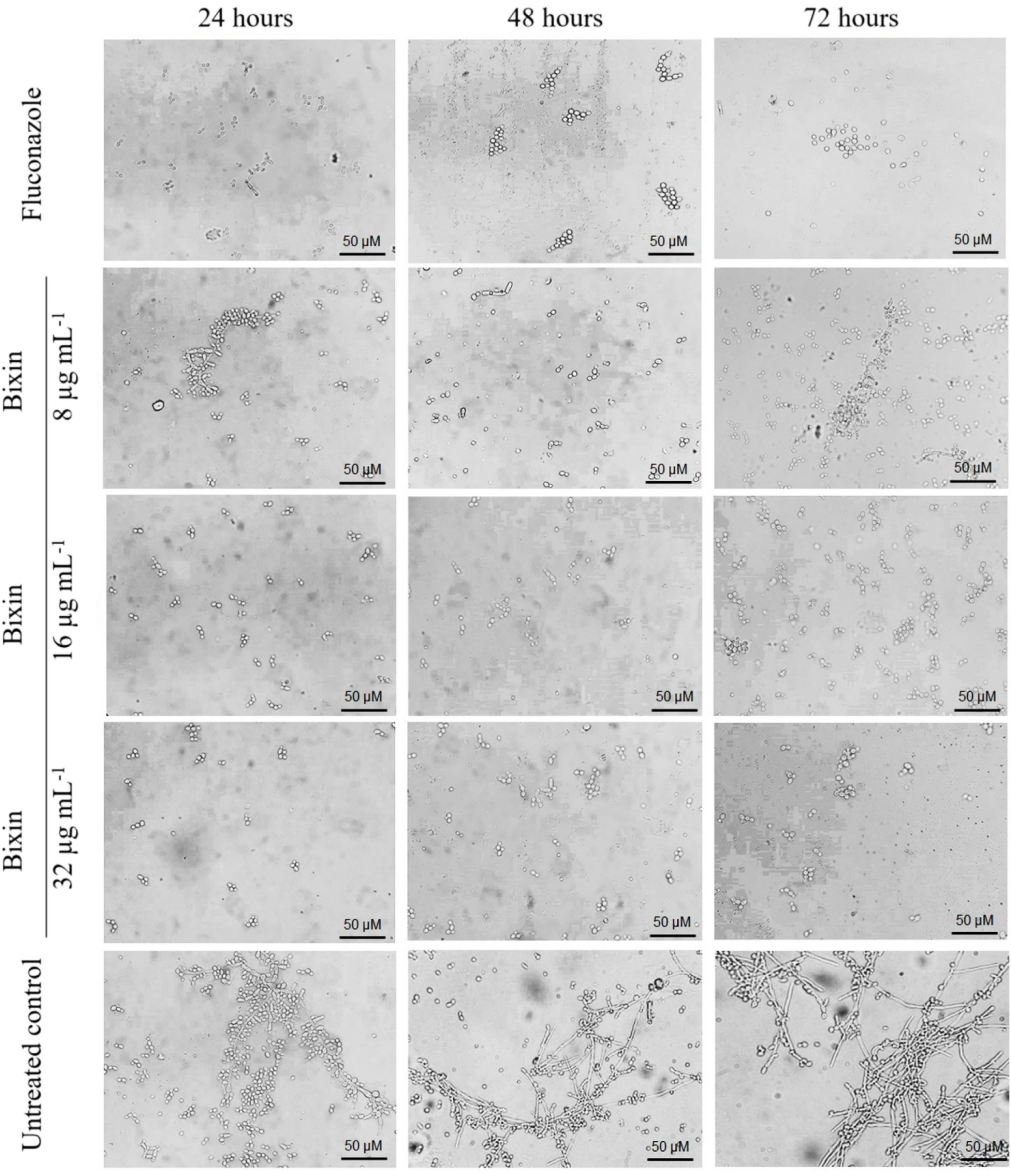
Effect of bixin (8 μg mL^–1^, 16 μg mL^–1^, and 32 μg mL^–1^) on the yeast-to-hypha transition in *C. albicans* ATCC 18804 incubated for 24, 48, and 72 h. Fluconazole (2 μg mL^–1^) was used as a positive control.

Some species of the *Candida* genus exhibit the ability to form biofilms, which constitutes an important virulence factor.^69^ Studies have shown that microorganisms are almost nonexistent in their free-floating planktonic form within host tissues; instead, they cluster together to form a multicellular community.^70^ Individual microorganisms within biofilms are incorporated into an extracellular polymeric matrix and characteristically display a phenotype that markedly differs from that of planktonic cells. Fundamentally, they are significantly less susceptible to antimicrobial agents, thus posing a substantial obstacle for the treatment of invasive candidiasis.^71,72^ Among the three classes of antifungal agents currently in clinical use, only amphotericin B and echinocandins, such as caspofungin, have demonstrated consistent *in vitro* activity against *C. albicans* biofilms,^73^ which justifies the search for new compounds with antibiofilm activity.

As previously shown (Table 3), purified bixin exhibits significant activity against planktonic cells of *C. glabrata* ATCC 2001 and *C. tropicalis* ATCC 28707 with MICs ≤ 4 μg mL^–1^. Although this action was not evaluated in this study, it likely extends to the sessile cells within biofilms formed by these species. To investigate the action of bixin on mature biofilms and given that this is a novel test, we developed biofilms of *C. albicans* ATCC 18804, with MIC and MFC of bixin determined to be 16 μg mL^–1^ and ≥ 1.024 μg mL^–1^, respectively. *C. albicans* is used as a model organism for studying fungal biofilms^74^ and remains the most commonly associated fungal species with biofilm formation, particularly on mucosal surfaces, epithelial cell linings, and implanted medical devices such as catheters, dental prosthetics, and heart valves.^75^

The picture representing the percentage reduction of mature biofilm of *C. albicans* ATCC 18804 in relation to bixin concentration and the positive control is shown in Figure 9.

**Figure 9:**
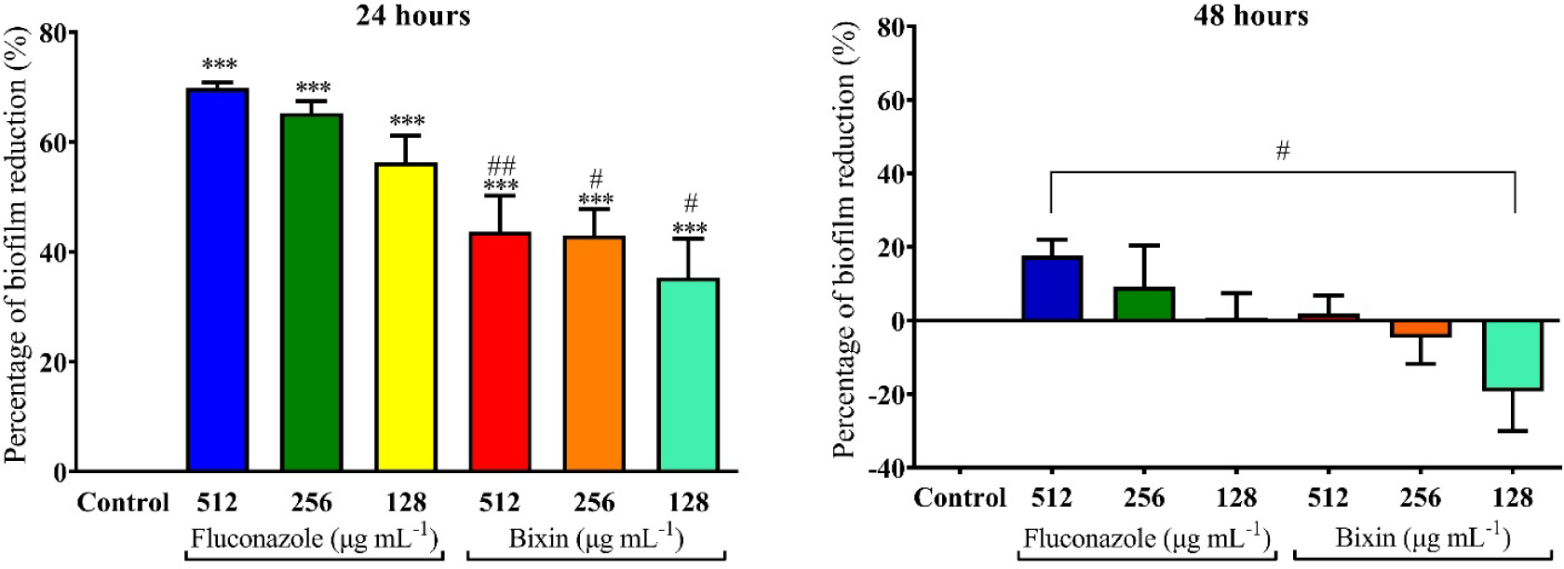
Graphical representation of the effect of bixin and fluconazole at concentrations of 16–512 μg mL^–1^ on mature biofilms formed by *C. albicans* ATCC 18804 after 24 and 48 h. Three asterisks (***) indicate a statistically significant difference from the control (*p* < 0.0001). The symbol (#) indicates a statistically significant difference from the wells treated with fluconazole (*p* < 0.01), and (##) indicates *p* < 0.001.

As observed, both fluconazole and bixin disrupt the mature biofilm after 24 h of incubation, starting at concentrations of 128 μg mL^–1^, compared to the untreated control. Although the effect of fluconazole is statistically greater than that of bixin (*p* < 0.0001), this result is noteworthy considering that bixin is a natural compound, readily available and widely explored for its medicinal properties.

In the context of natural compounds, eugenol (2000 μg mL^–1^) has been shown to promote a reduction of over 80% in the preformed biofilm of *C. albicans* ATCC 10231.^76,77^ Despite the high concentration, the authors concluded that this compound has potential therapeutic implications for candidiasis infections associated with biofilms. Teixeira (2014) also evaluated the effect of tyrosol, present in olive oil, on *Candida* spp. biofilms and observed a significant decrease in mature biofilm at a concentration of 700 μg mL^–1^, concluding that this compound has promising activity. Thus, the activity in disrupting the mature biofilm (∼50%) at lower concentrations (512 μg mL^–1^) than those reported in the cited studies should be considered an advantage in addition to the fungistatic action of bixin.^77^

Interestingly, after 48 h of incubation, the biofilm disruption effect was reduced for fluconazole and lost for bixin at the aforementioned concentrations. According to Mohamed (2016), this event occurs due to the growth of cells in a latent state present in the early phases of the biofilm.^78^ Therefore, in the first 24 h, the biofilm is more susceptible to antimicrobials, and after maturation, up to 48 h, it becomes increasingly tolerant due to the higher number of cells.^79^

#### 2.3.3 Toxicity and Selectivity Assays of Bixin

The assessment of the cytotoxicity of a compound with therapeutic potential is of extreme relevance as it reflects the safety of its use in vivo.^80^ The results for bixin are represented in Figure 10, where it can be observed that the substance exhibits cytotoxicity in a dose-dependent manner; that is, with increasing concentrations of bixin, there is a reduction in cell viability.

**Figure 10:**
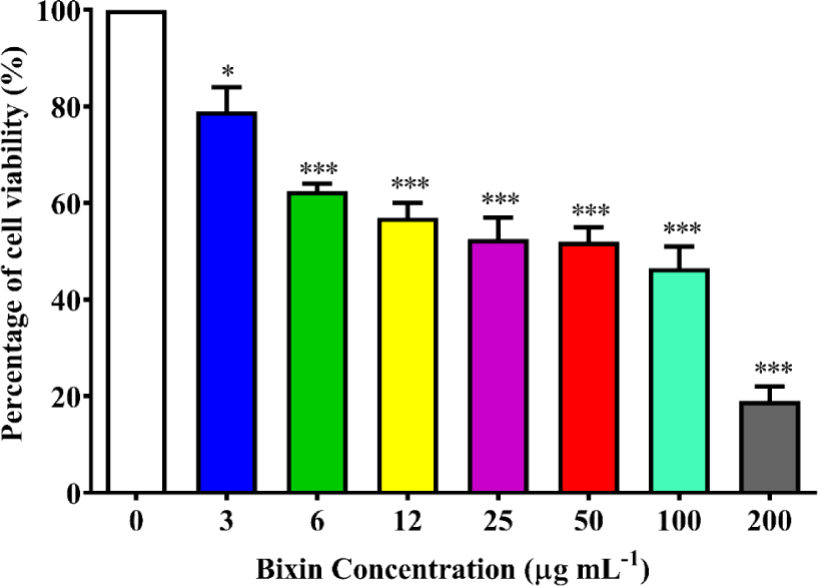
Graphical representation of the quantitative assessment of cell viability. % of viable cells observed in Vero cells after 48 h of bixin contact with the cell layer at concentrations of 0–200 μg mL^–1^. Three asterisks (***) indicate a statistically significant difference in the reduction of the % of viable cells compared to the concentration of 3 μg mL-1 (*p* < 0.0001).

Based on the results of the percentage of viable cells, the concentrations considered lethal for 50% of the cell population (CC50) were calculated, yielding values of 30.71 ± 0.037 μg mL^–1^ and 42.48 ± 1.16 μg mL^–1^ for bixin and fluconazole, respectively.^81^ The cytotoxic concentration found for bixin, when compared with curcumin (CC50 18 μg mL^–1^), another natural compound that exhibits dose-dependent toxicity,^82^ is promising and should be considered.

It is important to emphasize that cytotoxicity should not be evaluated in isolation when determining the therapeutic potential of a compound. In addition to cytotoxicity, the selectivity index (SI) should be determined for candidate compounds, as it reveals how targeted the compound is toward the intended pathogen in comparison to other components. Thus, the SI of an antimicrobial compound is calculated by considering the ratio of cytotoxicity (CC50) to the MIC determined for the microorganism, thereby expressing numerically how many times the compound is more selective for the pathogen of interest. According to Bézivin et al. (2003), SI values greater than 3 should arouse interest and prompt further study of the substance.^83^

The SI of bixin evaluated for yeast-like cells and Vero mammalian kidney cells is shown in Table 3. The results obtained for the studied *Candida* strains indicate that the selectivity of bixin is species-specific. It can be observed that bixin exhibits greater selectivity for *C. glabrata* ATCC 2001 (SI 15.36) and *C. tropicalis* ATCC 28707 (SI 7.68) compared to mammalian cells. This fact, along with the better MICs and death curve, reinforces the superior antifungal activity of bixin against these species.

The low selectivity indices obtained for bixin against other *Candida* species do not preclude the potential therapeutic use of the compound. For example, amphotericin B exhibits high cytotoxicity against microglial cells^63^ and is known for its nephrotoxicity;^84^ however, it remains part of the therapeutic protocol for severe fungal infections. Finally, with advancements in pharmaceutical technology, the availability of new pharmaceutical forms, such as nanotechnology,^85^ will enable the use of compounds considered “toxic” *in vitro* characterization assays, thus constituting a strategy for utilizing compounds with proven antifungal activity that are still considered of limited scale in the therapeutic arsenal.

## 3 Conclusion

In this study, we successfully developed, optimized, and validated a robust HPLC-UV method for the quantification of purified bixin, demonstrating high precision, accuracy, and selectivity. The extraction and purification procedures yielded bixin of high purity (>95%), predominantly in its natural 9’-cis configuration, as confirmed by FTIR, TG/DTA, and ^1^H NMR analyses. Notably, the purified compound exhibited moderate to significant antifungal activity against various *Candida* species, particularly *C. glabrata* and *C. tropicalis*, with favorable selectivity indices and fungistatic potential at clinically relevant concentrations. Bixin also demonstrated an inhibitory effect on virulence factors such as yeast-to-hypha transition and biofilm formation in *C. albicans*. Although the compound displayed dose-dependent cytotoxicity in Vero cells, its selectivity against key pathogenic yeasts supports its potential as a lead compound for antifungal drug development. Future studies focusing on formulation strategies, such as nanoencapsulation, and combination therapy may further enhance bixin’s therapeutic applicability and safety profile.

## 4 Experimental Section

### 4.1 Development and Validation of an HPLC Method for Bixin Analysis

The development and validation of HPLC method were carried out using a Shimadzu LC-10A integrated liquid chromatograph (Tokyo, Japan) equipped with a Shimadzu UV–vis detector and a reversed-phase C18 column (250 mm × 4.6 mm, 5 μm particle size). The detector wavelength was set to 470 nm, and the column temperature was maintained at 40 °C. The mobile phase consisted of acetonitrile and an HPLC-grade aqueous solution containing 2% (v/v) glacial acetic acid, delivered in isocratic mode. Prior to use, the mobile phase was vacuum-filtered through a 0.45 μm membrane and sonicated in a bath for 15 min. The flow rate was adjusted to 1.2 mL min^–1^, and the injection volume was 20 μL. Data acquisition and processing were performed using LC Solution software (Version 1.25, Shimadzu, Tokyo, Japan).

According to the ICH Q2/R2 guidelines (2024), the reliability and consistency of the method were assessed by evaluating specificity, linearity, accuracy, repeatability, intermediate precision, detection limit, quantitation limit, and system suitability. All tests were performed in compliance with the relevant regulatory requirements. Primary standard solutions of bixin (Sigma-Aldrich, USA) at a concentration of 1000 μg mL^–1^ were prepared in ethanol. These solutions were protected from light by wrapping them in aluminum foil and were stored in amber glass containers at 4 °C (±0.5 °C) until analysis. Secondary standard solutions were obtained by serial dilution of the primary solution using acetonitrile as the diluent. All solutions were filtered through a 0.45 μm syringe filter (Kasvi, Brazil) before analysis.

The specificity of the method was verified using common pharmaceutical excipients dissolved in acetonitrile. The excipients tested included polyvinylpyrrolidone (Synth, Brazil), poloxamer 407 (Sigma-Aldrich, USA), hydroxypropyl methylcellulose (Infinity Pharma, Brazil), glycerol (Synth, Brazil), Eudragit RL 100 (Evonik, Germany), and Eudragit RS 100 (Evonik, Germany). The resulting chromatograms were examined to detect any potential interfering peaks at the retention time or in the peak area of the analyte.

To evaluate linearity, bixin was accurately weighed and dissolved in ethanol to prepare a primary stock solution at 1000 μg mL^–1^. From this, working standard solutions were obtained at concentrations of 12.5, 25.0, 37.5, 50.0, 62.5, and 75.0 μg mL^–1^ through successive dilutions. Each concentration was injected in triplicate, and the peak areas were recorded to construct a calibration curve by plotting concentration against detector response. Outliers were identified and removed using the standardized Jackknife residual test.^86^ To confirm the normality of residuals, a normal probability plot and the Ryan-Joiner correlation coefficient were applied.^87^ Residual autocorrelation was visually inspected, and the Durbin-Watson statistic was used to evaluate their independence.^88^ Homoscedasticity was tested using the modified Levene test.^89^ A new calibration curve was then constructed, and the regression equation was calculated using the ordinary least-squares (OLS) method. The coefficient of determination (R^2^) was obtained, and the adequacy of the linear model was verified by analysis of variance (ANOVA) for lack-of-fit (*p* > 0.05) and significance testing of the regression parameters (*p* > 0.05). Once linearity was confirmed, the slope and intercept of the calibration curve were determined.

The precision of the method was assessed in terms of both repeatability (intraday precision) and intermediate precision (interday precision). Repeatability was evaluated by analyzing bixin samples at three different concentrations (25.0, 50.0, and 75.0 μg mL^–1^) in triplicate within a single day. Intermediate precision was assessed by preparing and analyzing the same concentrations over three consecutive days, with nine determinations in total. For both repeatability and intermediate precision, results were expressed as the relative standard deviation (%RSD) of the peak areas.

Accuracy was evaluated at the same three concentration levels: 25.0, 50.0, and 75.0 μg mL^–1^. For each level, three replicates were independently prepared and analyzed. Accuracy was expressed as the recovery percentage, calculated by the ratio between the experimentally determined mean concentration and the corresponding theoretical value.

The sensitivity of the method was determined by calculating the limit of detection (LOD) and the limit of quantification (LOQ). These values were derived from three calibration curves generated during the linearity tests. The LOD was calculated as 3.3 times the standard deviation of the y-intercept (b) divided by the mean slope (a) of the calibration curves. The LOQ was similarly calculated, using a factor of 10 times the standard deviation of the y-intercept divided by the mean slope. These parameters established the minimum concentrations of bixin that could be reliably detected and quantified using the validated HPLC method.

### 4.2 Isolation and Purification of Bixin from *B. orellana* L. Seeds

Six samples of annatto seeds (*B. orellana* L.), approximately 200 g each, were obtained from the Central Market of Divinópolis (MG, Brazil). The seeds were wrapped in qualitative filter paper (Synth, Brazil) and subjected to organic extraction using a Soxhlet apparatus (Diogolab, Brazil). Sequential extractions were performed initially with hexane (Synth, Brazil) followed by chloroform (Cromato, Brazil).^90^ The solvents were evaporated under reduced pressure using a rotary evaporator (Model RV10 Digital V., Ika, Brazil), yielding a solid bixin extract. This extract was further purified by recrystallization in acetone (Synth, Brazil). The purified bixin crystals were dried under vacuum, collected in an amber vial, and stored under refrigeration at −6 °C.

### 4.3 Physicochemical Characterization of Purified Bixin

The bixin characterized in this study was extracted, purified, and recrystallized from *B. orellana* L. seeds. Thermal Analysis (TG/DTA) curves were simultaneously performed in a Thermogravimetric Analyzer (TGA) SDT-Q600 (TA Instruments, New Castle, DE, USA) under dynamic nitrogen atmosphere, with a flow rate of approximately 50 mL min^–1^. About 5 mg of the sample were added to alumina crucibles and heated from 20 to 350 °C at a heating rate of 10 °C min^–1^. The equipment was previously calibrated with indium (melting point 156.6 °C; ΔH = 28.54 J/g) and lead (melting point 327.5 °C). The obtained data were analyzed with TA Instruments Universal Analysis 2000 software and were later plotted using Origin Pro 8.0 software package (OriginLab Corporation, USA).

Attenuated total reflectance Fourier transform infrared (ATR-FTIR) spectroscopy was executed using an ATR-Spectrum One spectrometer (PerkinElmer, Massachusetts, IL, USA) over the 4000–650 cm^–1^ wavenumber range. The obtained spectra are the result of the average of 32 scans performed with a resolution of 4 cm^–1^.

The Nuclear Magnetic Resonance (NMR) spectra were recorded on a Bruker DPX-400 Avance (400 MHz) spectrometer (Bruker Corporation, Massachusetts, IL, USA). The CDCl_3_ solvent used had a minimum isotopic purity of 99.5% D (Sigma-Aldrich, USA). The samples were prepared in NMR tubes with a length of 8.00 in. and an outer diameter of 5 mm. The one-dimensional ^1^H NMR experiments were performed using a 5 mm dual ^1^H/^13^ C direct detection probe. The mixing time was 500 ms. The analysis was conducted at 27 °C.

### 4.4 *In Vitro* Assessment of the Antifungal Activity of Bixin

#### 4.4.1 Yeasts Samples

The antifungal activity of purified bixin was assessed against several species obtained from the American Type Culture Collection (ATCC): *Candida albicans* ATCC 26790, *Candida albicans* ATCC 14053, *Candida albicans* ATCC 18804, *Candida glabrata* ATCC 2001, *Candida krusei* ATCC 6258, *Candida krusei* ATCC 34135, *Candida parapsilosis* ATCC 22019, and *Candida tropicalis* ATCC 28707. These strains were provided by the Reference Microorganisms Laboratory of the Oswaldo Cruz Foundation (FIOCRUZ), Rio de Janeiro, Brazil. Yeasts were stored at −80 °C in Sabouraud-Dextrose Broth (SDB) (Himedia, India) containing 20% glycerol (Dinâmica, Brazil) as a cryoprotectant. For the experiments, the microorganisms were reactivated in SDB and incubated at 35 °C for 48 h. Following incubation, each microorganism was streaked on Sabouraud-Dextrose Agar (SDA) (Acumedia, USA) and incubated at 35 °C for 24–48 h before use in the antifungal assays.

#### 4.4.2 *In Vitro* Antifungal Susceptibility Testing

The qualitative antifungal activity of purified and crystallized bixin was evaluated using the disk diffusion method, following the Clinical and Laboratory Standards Institute (CLSI) guidelines M44.^91^ An inoculum of each yeast was prepared in 0.85% sterile saline solution (NaCl, Synth, Brazil) from cultures grown on SDA (Acumedia, USA) for 48 h. The turbidity of the suspension was adjusted in a spectrophotometer (Nova Instruments, Brazil) at an optical density of 530 nm, equivalent to the 0.5 McFarland standard, corresponding to approximately 10^6^ CFU mL^–1^.

Previously sterilized 6 mm qualitative filter paper discs (Synth, Brazil) were impregnated with bixin solutions diluted in dimethyl sulfoxide (DMSO, Chromato, Brazil) at concentrations of 10, 25, and 190 μg. The antimicrobials fluconazole (Fagron, Brazil), nystatin (Pharma Nostra, Brazil), and amphotericin B (Inlab, Brazil) were used as positive controls. Fluconazole was diluted in water, while the other compounds were diluted in DMSO, with concentrations based on reference standards for each experiment.^91^ In addition, DMSO was used as a negative control, and a blank disc without any substance served as the sterility control.

Inhibition zone diameters of fluconazole were classified as follows for *Candida* spp.: ≥ 19 mm (sensitive), 15–18 mm (dose-dependent), and ≤ 14 mm (resistant).^91^ For amphotericin B, inhibition zones of ≥ 15 mm were considered sensitive, 10–14 mm dose-dependent, and ≤ 10 mm resistant.^40,41^

The minimum inhibitory concentration (MIC) of bixin for the *Candida* species included in this study was determined using the broth microdilution method, following CLSI documents M27-A3 ^92^ and M07,^93^ with modifications as described by Lima et al. (2019).^94^

Purified bixin concentrations ranged from 1 to 1024 μg mL^–1^, while control antifungal concentrations were 0.125–64 μg mL^–1^ for fluconazole, 0.313–16 μg mL^–1^ for amphotericin B ^91^, and 0.015–8 μg mL^–1^ for nystatin.^95^ DMSO was used as the solvent control. All tests were conducted in triplicate across two independent experiments. Plates were incubated at 35 °C for 48 h, and MIC values were determined by visual inspection of the wells, confirmed using images captured with a Motic photomicroscope BA 310. The MIC of bixin was defined as the lowest concentration that inhibited 50% of fungal growth compared to the untreated control.

The fungicidal activity of purified bixin was evaluated by determining the minimum fungicidal concentration (MFC).^96^ Briefly, 10 μL from optically clear wells in the MIC assay were aliquoted and dispensed onto the surface of SDA. The material was spread using the plate-spreading technique, and plates were incubated at 35 °C for 48 h. MFC was defined as the lowest concentration that inhibited 99% of colony growth compared to the untreated control. The solvent control (DMSO) was also evaluated. All experiments included controls for medium sterility and microorganism growth.

#### 4.4.3 Time-Kill Curve

The evaluation of fungal growth kinetics (*C. albicans* ATCC 10231 and *C. tropicalis* ATCC 28707) in the presence of bixin was conducted using a time-kill curve assay as described by Zore et al. (2011),^97^ with modifications. An inoculum of the studied species was prepared to achieve a cell density of 10^6^ CFU mL^–1^, as previously outlined. In a test tube containing 9 mL of SDB, 1 mL of the inoculum was added, resulting in a final cell density of 10 ^5^ CFU mL^–1^. Bixin was added at a concentration of 1024 μg mL^–1^ (the minimum fungicidal concentration for the studied species).

The tubes were incubated at 35 °C, and 100 μL aliquots were collected at 0, 2, 4, 6, 8, 10, 12, 24, 36, and 48 h. These aliquots were plated on SDA supplemented with chloramphenicol (50 mg mL^–1^, Acumedia, Brazil) using the spread plate technique. All samples were plated directly from the initial tube (10 ^5^ CFU mL^–1^) and from four serial dilutions (10^–4^, 10^–3^, 10^–2^, 10^–1^). The plates were incubated for 48 h at 35 °C, after which colonies were counted, and the calculation of CFU mL^–1^ was performed.^97^

#### 4.4.4 Antivirulence Factor Activity of Bixin

##### 4.4.4.1 *Candida* Yeast-to-Hyphal Transition Inhibition Assay

The effect of bixin (1/2 MIC, MIC, and 2× MIC, respectively, 8 μg mL^–1^, 16 μg mL^–1^, and 32 μg mL^–1^) and fluconazole (2 μg mL^–1^) on the yeast-to-hyphae transition in *C. albicans* ATCC 18804 was evaluated as described by Lima et al. (2019).^94^ Hyphal induction was performed by incubating *C. albicans* (10^3^ CFU mL^–1^) in microplates containing fetal bovine serum (FBS, Sigma-Aldrich, USA) supplemented with bixin. The microplates were incubated at 35 °C for 48 h, followed by the aspiration of 20 μL from each well to prepare fresh slides, which were then evaluated by light microscopy (Zeiss, Switzerland). The qualitative results of bixin’s effect on the yeast-to-hypha transition were compared with the untreated control and the positive control (fluconazole). The experiment was conducted in duplicate and independently.

##### 4.4.4.2 Effect on Mature Biofilm

The effect of bixin (16–512 μg mL^–1^) on mature biofilm was assessed using the crystal violet method as described by Lima et al. (2019).^94^ The inoculum of *C. albicans* ATCC 18804 with a cell density of 10^7^ CFU mL^–1^ was prepared as described above. Subsequently, 100 μL of the inoculum was transferred to 10 mL of SDB (Himedia, India) supplemented with 100 μM glucose (Synth, Brazil). From this suspension, 100 μL was aliquoted and transferred to microplates, which were then incubated at 37 °C for 48 h to allow for biofilm formation and adhesion. After incubation, the supernatant was discarded, and the planktonic cells were removed with two washes using a 0.85% NaCl solution (Synth, Brazil).

Next, 100 μL of SDB containing varying concentrations of bixin were added over the mature biofilms, followed by further incubation for 24 and 48 h at 37 °C. Finally, the supernatant was removed, and the microplates were washed with a 0.85% NaCl solution (twice). For biofilm assessment, 125 μL of 0.1% crystal violet (Sigma-Aldrich, USA) were applied to the wells, and the microplates were incubated for 15 min at room temperature. The supernatant was then discarded, and excess dye was removed by washing with 0.85% NaCl solution. The microplates were allowed to dry inverted at room temperature for 1 h. Subsequently, the dye was solubilized with 125 μL of 95% ethanol, and absorbance was measured using a spectrophotometer at 550 nm.^98^ The results were expressed graphically as the percentage of biofilm reduction relative to the untreated control, with fluconazole used as a positive control at the same concentrations as bixin.

#### 4.4.5 Toxicity and Selectivity Assays of Bixin

##### 4.4.5.1 Cytotoxicity Assessment

Cytotoxicity assays for bixin were conducted using Vero ATCC CCL-81 cells, as recommended by ISO 10993–5.^99^ Cell culture was performed in 75 mL bottles containing Dulbecco’s Modified Eagle Medium (DMEM; Cultilab, Brazil) supplemented with 5% FBS, 50 mg mL^–1^ of l-glutamine (Sigma-Aldrich, USA), and 0.3% of a penicillin-streptomycin-amphotericin B solution (10,000 IU mL^–1^ + 10 mg mL^–1^ + 2 mg mL^–1^) (Sigma-Aldrich, USA).^100^ The bottles were incubated at 37 °C in a 5% CO_2_ atmosphere.

Following cell culture, cell microplates were prepared by adding 2.5 × 10^4^ to 3.0 × 10^4^ cells per well, followed by the addition of 100 μL of DMEM medium supplemented with 5% FBS. The plates were incubated at 37 °C in a 5% CO_2_ atmosphere for 24 h. For Vero cell treatment, bixin was diluted in DMEM in another microplate at concentrations ranging from 0 to 200 μg mL^–1^. Then, 100 μL of DMEM medium and 100 μL of the diluted compounds from the mirror plate were added to the cell monolayer and incubated at 37 °C in a 5% CO_2_ atmosphere for 48 h.

Finally, the cytotoxicity of the compounds was determined using the colorimetric method with 3-(4,5-dimethylthiazol-2-yl)-2,5-diphenyltetrazolium (MTT; Sigma-Aldrich, USA).^101^ In viable cells, mitochondrial dehydrogenases convert MTT into insoluble purple crystals. After solubilizing the crystals in DMSO, cell viability was measured spectrophotometrically at 540 nm. From the cell viability values, the cytotoxic concentration for 50% of the cells (CC_50_) was calculated using eq 1:

1 $$CC_{50}=\left(\frac{B}{A}\right)\times 100$$

where A and B represent the optical density (OD) at 540 nm of the wells containing untreated cells (A) and treated cells (B), respectively.^81,96^

##### 4.4.5.2 Determination of the Selectivity Index

To determine the selectivity index of the compound for yeast samples in relation to mammalian cells (Vero), the methodology described by Lyu et al. (2016) was employed.^96^ The selectivity index for bixin and fluconazole was calculated by dividing the CC_50_ obtained with Vero cells by the MIC of each *Candida* strain. The resulting value represents the “X”-fold selectivity of the compound for fungal cells relative to mammalian cells.

### 4.5 Statistical Analysis

All assays were performed in triplicate. Statistical analysis was conducted using GraphPad Prism 8 software (GraphPad Software, Inc., USA). Data are expressed as mean ± standard deviation of the mean. Statistical significance was assessed using Two-Way ANOVA, with *p* < 0.05 considered significant.

## Abbreviations

ANOVA: Analysis of Variance
ATR-FTIR: Attenuated Total Reflectance Fourier Transform Infrared
ATCC: American Type Culture Collection
CC50: 50% Cytotoxic Concentration
CFU: Colony Forming Units
DAD: Diode Array Detector
DMEM: Dulbecco’s Modified Eagle Medium
DMSO: Dimethyl Sulfoxide
DTA: Differential Thermal Analysis
FBS: Fetal Bovine Serum
FTIR: Fourier Transform Infrared
HPLC: High-Performance Liquid Chromatography
ICH: International Council for Harmonisation
MFC: Minimum Fungicidal Concentration
MIC: Minimum Inhibitory Concentration
MTT: 3-(4,5-dimethylthiazol-2-yl)-2,5-diphenyltetrazolium
NMR: Nuclear Magnetic Resonance (^1^H NMR: Proton Nuclear Magnetic Resonance)
RSD: Relative Standard Deviation
SDA: Sabouraud-Dextrose Agar
SDB: Sabouraud-Dextrose Broth
SI: Selectivity Index
TG: Thermogravimetric
TG/DTA: Thermogravimetric/Differential Thermal Analysis
UV–vis: Ultraviolet–Visible.
